# Dexamethasone inhibits endotoxin‐induced coagulopathy in human lungs

**DOI:** 10.1111/jth.13504

**Published:** 2016-10-24

**Authors:** J. Bartko, C. Schoergenhofer, M. Schwameis, N. Buchtele, J. Wojta, G. Schabbauer, L. Stiebellehner, B. Jilma

**Affiliations:** ^1^ Department of Clinical PharmacologyMedical University of ViennaViennaAustria; ^2^ Department of Internal Medicine IIMedical University of ViennaViennaAustria; ^3^ Institute of Physiology, Center for Physiology and PharmacologyMedical University of ViennaViennaAustria

**Keywords:** acute respiratory distress syndrome, coagulation, dexamethasone, lung, pneumonia

## Abstract

**Essentials**

- Glucocorticoids are associated with an increased risk of thrombosis.
- Healthy volunteers received dexamethasone or placebo in an endotoxin lung instillation model.
- Dexamethasone suppressed thrombin generation in bronchoalveolar lavage.
- Glucocorticoids inhibit endotoxin induced pulmonary coagulopathy.

**Summary:**

**Background**

Activation of local and systemic coagulation is a common finding in patients with pneumonia. There is evidence that glucocorticoids have procoagulant activity in the circulation, particularly in the context of inflammation. The effects of glucocorticoids on local pulmonary coagulation have not yet been investigated.

**Objective**

To use a human model of lung inflammation based on the local instillation of endotoxin in order to investigate whether glucocorticoids alter pulmonary coagulation.

**Methods**

Twenty‐four healthy volunteers were randomized to receive either dexamethasone or placebo in a double‐blind trial. Endotoxin was instilled via bronchoscope into right or left lung segments, followed by saline into the contralateral site. Six hours later, a bilateral bronchoalveolar lavage (BAL) was performed and coagulation parameters were measured.

**Results**

Endotoxin induced activation of coagulation in the bronchoalveolar compartment: the level of prothrombin fragment 1 + 2 (F_1 + 2_) was increased three‐fold (248 pmol L^−1^, 95% confidence interval [CI] 43–454 versus 743 pmol L^−1^, 95% CI 437–1050) and the level of thrombin–antithrombin complex (TATc) was increased by ~ 50% (31 μg L^−1^, 95% CI 18–45 versus 49 μg L^−1^, 95% CI 36–61) as compared with saline‐challenged segments. Dexamethasone reduced F_1 + 2_ (284 pmol L^−1^, 95% CI 34–534) and TATc (9 μg L^−1^, 95% CI 0.7–17) levels almost to those measured in BAL fluid from the saline‐instilled segments in the placebo group. Dexamethasone even profoundly reduced F_1 + 2_ levels (80%) in saline‐instilled lung segments (50 pmol L^−1^, 95% CI 12–87). In contrast, dexamethasone had no effect on systemic F_1 + 2_ levels.

**Conclusions**

Dexamethasone inhibits endotoxin‐induced coagulopathy in lungs. This trial is the first to provide insights into the effects of glucocorticoids on pulmonary coagulation in response to endotoxin.

## Introduction

Activation of local and systemic coagulation is a common finding in patients with severe pneumonia 1, 2. In the lung, activation of coagulation is initiated through formation of a tissue factor (TF)–coagulation factor VII complex, which leads to thrombin generation and subsequently to fibrin formation 2, 3. Intra‐alveolar fibrin deposition keeps the pathogen from spreading into the circulation, and serves as a matrix for tissue repair 4, 5. However, severe inflammation results in excessive fibrin formation, which can progress to severe respiratory failure, as seen in patients with acute respiratory distress syndrome (ARDS) 6. Patients with lower inflammatory cytokine levels have a better outcome 7, so reduction of inflammation has become a major drug target. The therapeutic potential of glucocorticoids as adjunctive therapy in pneumonia or ARDS has been investigated, but the results remain controversial 8, 9, 10, 11. Glucocorticoids are linked to an increased risk of thrombosis 12, and there is evidence that glucocorticoids have procoagulant activity in the circulation 13, particularly in the context of inflammation 14, 15. The effects of glucocorticoids on local pulmonary coagulation have not yet been investigated. We therefore used a human lung inflammation model based on the local instillation of endotoxin to study the effects of glucocorticoids on coagulation markers in bronchoalveolar lavage (BAL) fluid (BALF).

## Materials and methods

### Trial design

**Figure 1:**
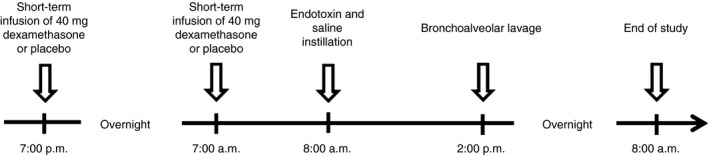
Experimental design.

The trial was performed concurrently with an investigation on the anti‐inflammatory effects of dexamethasone published recently 16. This randomized, double‐blind and placebo‐controlled trial was conducted at the Department of Clinical Pharmacology, Medical University of Vienna, Austria between July 2011 and June 2012. The trial was approved by the institutional review ethics board (Ethics Committee of the Medical University of Vienna), and was registered at the clinical trial registry http://www.ClinicalTrials.gov (identifier: NCT01714427). Randomization was performed with an open‐access randomization generator (http://www.randomization.com). Two sets of sealed codes/labels with the randomization number containing information about the treatment allocation for the particular subject were prepared for each subject. Randomization was performed by the use of sealed opaque envelopes, which were produced before the start of the study by a staff member not otherwise involved in the study. The trial was conducted in accordance with the Declaration of Helsinki, and informed consent was given by all study participants before trial entry. The major eligibility criteria for inclusion of participants were that they should be healthy male or female volunteers aged 19–40 years and be non‐smokers. Nine women and 15 men were randomly assigned to receive either two infusions of 40 mg of dexamethasone (Merck, Vienna, Austria) separated by 12 h (total dose: 80 mg) or equivalent placebo (physiologic saline) in a double‐blind design (Fig. 1). After the second infusion, subjects were premedicated with 12.5 mg of dihydrocodein (Teofarma, Valle Salimbene, Italy) and underwent the first bronchoscopy. Subjects received midazolam (median dose: 10.5 mg; Roche, Vienna, Austria) and 100 mL of propofol (1%; AstraZeneca, Vienna, Austria) intravenously for sedation. Lidocaine (AstraZeneca) was used topically for airway anesthesia. A balloon‐tipped monitoring catheter (Swan‐Ganz monitoring catheter; Edwards Lifesciences, Irvine, CA, USA) was inserted through a flexible fiberoptic video bronchoscope (model EB‐1970K or EB‐1570K; Pentax Medical Europe, Hamburg, Germany) into a lung subsegment (middle lobe or lingula). After inflation of the balloon, 10 mL of prewarmed sterile isotonic saline was instilled, followed by 10 mL of air, and the catheter was kept in place for 2 min. Subsequently 4 ng kg^−1^ body weight of National Reference Endotoxin (*Escherichia coli* O:113, CC‐RE‐Lot 3, NIH, dissolved in 2 mL of saline), followed by 10 mL of saline and 10 mL of air, was instilled into the contralateral lung in the same way. Instillation of endotoxin through a bronchoscope induces an interleukin‐6‐driven inflammatory and procoagulant response in the bronchoalveolar compartment 17, 18, 19, 20, 21. Bronchoscopy was followed by 30° head of the bed elevation. Throughout the first 5 h after endotoxin instillation, all subjects were confined to bed rest, and vital parameters were monitored continuously with an automated monitoring system (Care View System; Hewlett Packard, Böblingen, Germany). Concurrently, physiologic saline (200 mL h^−1^) was administered to all subjects to maintain adequate hydration. After 6 h, BAL was performed at each lung site. Aliquots of 20–40 mL of prewarmed saline (total volume: 140 mL) were instilled. The fluid was retrieved by syringe and suction, with avoidance of excessive negative suction pressures. Plasma samples were collected at – 13 h, – 1 h, 6 h and 24 h relative to endotoxin challenge.

### Assays

After collection, BALFs were immediately put on ice and then centrifuged for 10 min at 900 ×*g* at 4 °C. The supernatant was aliquoted and stored at − 80 °C until assays were performed. The levels of prothrombin fragment 1 + 2 (F_1 + 2_), thrombin–antithrombin complex (TATc), soluble TF (sTF) antigen, plasminogen activator inhibitor 1 (PAI‐1) and tissue‐type plasminogen activator (t‐PA) were measured with specific enzyme immunoassays, according to the manufacturers’ instructions (F_1 + 2_, Enzygnost F_1 + 2_ [Siemens, Marburg, Germany]; TATc, Enzygnost TAT micro [Siemens]; sTF, Imubind Tissue Factor ELISA [Sekisui Diagnostics, Stamford, CT, USA]; PAI‐1 activity, Technozym PAI‐1 Actibind ELISA [Technoclone, Vienna, Austria]; PAI‐1 antigen, Quantikine Human Total Serpin E1/PAI‐1 Immunoassay [R&D Systems, Minneapolis, MN, USA]; and t‐PA antigen and activity, Technozym t‐PA Combi Actibind ELISA [Technoclone]). The F_1 + 2_, TATc and sTF assays are sensitive to detect even low values in plasma and BALF of healthy volunteers as previously described 22, 23, 24. The lower limits of quantification were 20 pmol L^−1^ for F_1 + 2_, 2 μg L^−1^ for TATc, 0.05 ng mL^−1^ for sTF, 0.49 IU mL^−1^ for PAI‐1 activity, 0.313 ng mL^−1^ for PAI‐1 antigen, 0.05 IU mL^−1^ for t‐PA activity and 0.1 ng mL^−1^ for t‐PA antigen). Fibrinogen was measured with the Clauss method in an accredited routine laboratory.

### Statistical analysis

The original sample size calculation was based on interleukin‐6 levels in BALF 17. For this part of the analysis, an additional sample size calculation for the coagulation marker F_1 + 2_ was performed. On the basis of a previous publication, we estimated that the standard deviation of F_1 + 2_ would be similar to the mean F_1 + 2_ levels in BALF (0.6 nmol L^−1^ in patients suffering from pneumonia and 0.15 nmol L^−1^ in controls) 18. We calculated that we could detect a 125% difference between treatments and baseline. This was deemed to be adequate, in view of a four‐fold higher F_1 + 2_ level in patients with pneumonia than in healthy controls 18. Values are expressed as mean and standard error of the mean unless otherwise noted. A repeated measures anova was followed by non‐parametric tests because of a non‐normal distribution of data. Statistical comparisons between groups were performed with the Mann–Whitney *U*‐test, and comparisons between lung sites of subjects were performed with the Wilcoxon test. The median obtained lavage volumes were comparable between lung sites: endotoxin 45 mL (interquartile range [IQR] 35–50 mL) and saline 54 mL (IQR 39–59 mL) in the placebo group; endotoxin 49 mL (IQR 43–64 mL) and saline 53 mL (IQR 45–61 mL) in the dexamethasone group 16. We performed a sensitivity analysis adjusting for differences in the BALF recovered, although the value of this is debatable, because the dilution is probably always the same after instillation of 140 mL of saline. The results did not differ markedly when the *P*‐values of the sensitivity analyses or the unadjusted *P*‐values were used (Table S1). Statistical calculations were performed with commercially available statistical software (statistica Version 6.1; Stat Soft, Tulsa, OK, USA).

## Results

Endotoxin instillation into the lung induced a mild systemic inflammatory response in placebo‐treated individuals, and was generally well tolerated, without the occurrence of any severe adverse events; this has been reported previously, because the trial was performed concurrently with an investigation on the anti‐inflammatory effects of dexamethasone 16. The most pronounced effect of dexamethasone was complete prevention of the endotoxin‐induced protein extravasation into the alveolar space.

### Effects of endotoxin challenge on bronchoalveolar coagulation

**Figure 2:**
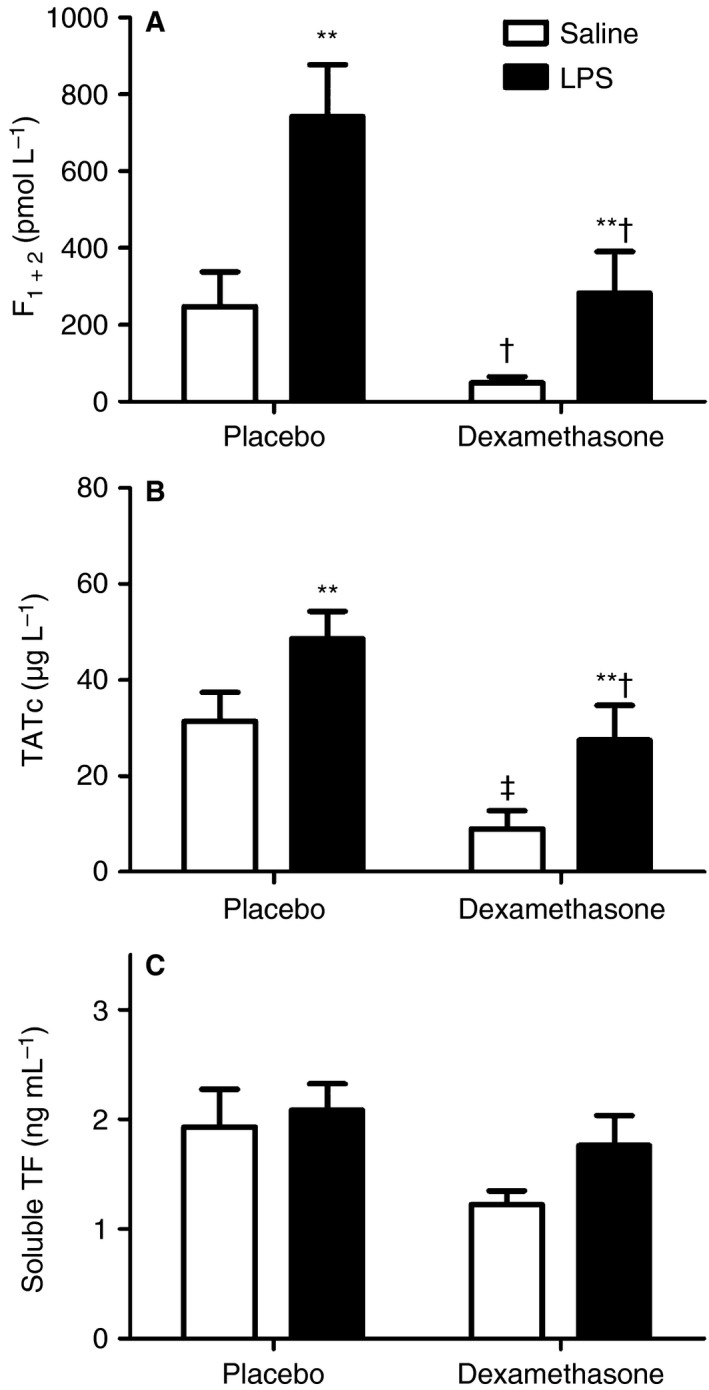
Bronchoalveolar lavage fluid (BALF) from 24 healthy volunteers who received dexamethasone (*n* = 11) or placebo (*n* = 13). Lipopolysaccharide (LPS) (4 ng kg^−1^) was instilled randomly via bronchoscopy into a left or a right lung segment. BALF samples were obtained 6 h after LPS instillation. Dexamethasone reduced BALF levels of prothrombin fragment 1 + 2 (F_1 + 2_) (A) and thrombin–antithrombin complex (TATc) (B) in LPS‐challenged and saline‐challenged lung segments as compared with placebo. Dexamethasone had no significant effect on soluble tissue factor (TF) (C). Data represent means ± standard errors of the mean. **P < 0.01 versus saline; †*P* < 0.05 and ‡*P* < 0.01 for comparison between dexamethasone and placebo treatment.

Instillation of 4 ng kg^−1^ lipopolysaccharide (LPS) increased BALF F_1 + 2_ levels three‐fold (*P* = 0.007; Fig. 2A) as compared with BALF from saline‐instilled (contralateral) lung sites. Similarly, TATc levels increased by 50% (*P* = 0.005; Fig. 2B) in BALF samples from LPS‐challenged lungs in comparison with BALF samples from the control segments.

### Dexamethasone effects on bronchoalveolar coagulation

F_1 + 2_ levels in BALF from LPS‐challenged lungs were approximately three‐fold higher with placebo than with dexamethasone (*P* = 0.02; Fig. 2A). Analogously, TATc levels were 40% lower in the dexamethasone group (*P* = 0.04; Fig. 2B). Dexamethasone reduced thrombin formation even in saline‐instilled segments (F_1 + 2_ levels [*P* = 0.01; Fig. 2A] by 80%, and TATc levels by 70% [*P* = 0.005; Fig. 2B] as compared with placebo). Endotoxin increased sTF levels in BALF minimally; the difference was not significant (*P* = 0.1 for dexamethasone, and *P* = 0.5 for placebo; Fig. 2C). sTF levels did not differ significantly between the groups (*P* = 0.16 for saline and *P* = 0.37 for LPS; Fig. 2C). BALF levels of PAI‐1 activity and antigen and of t‐PA activity and antigen were below the lower limits of quantification (data not shown).

### Systemic response to endotoxin and effects of dexamethasone

**Figure 3:**
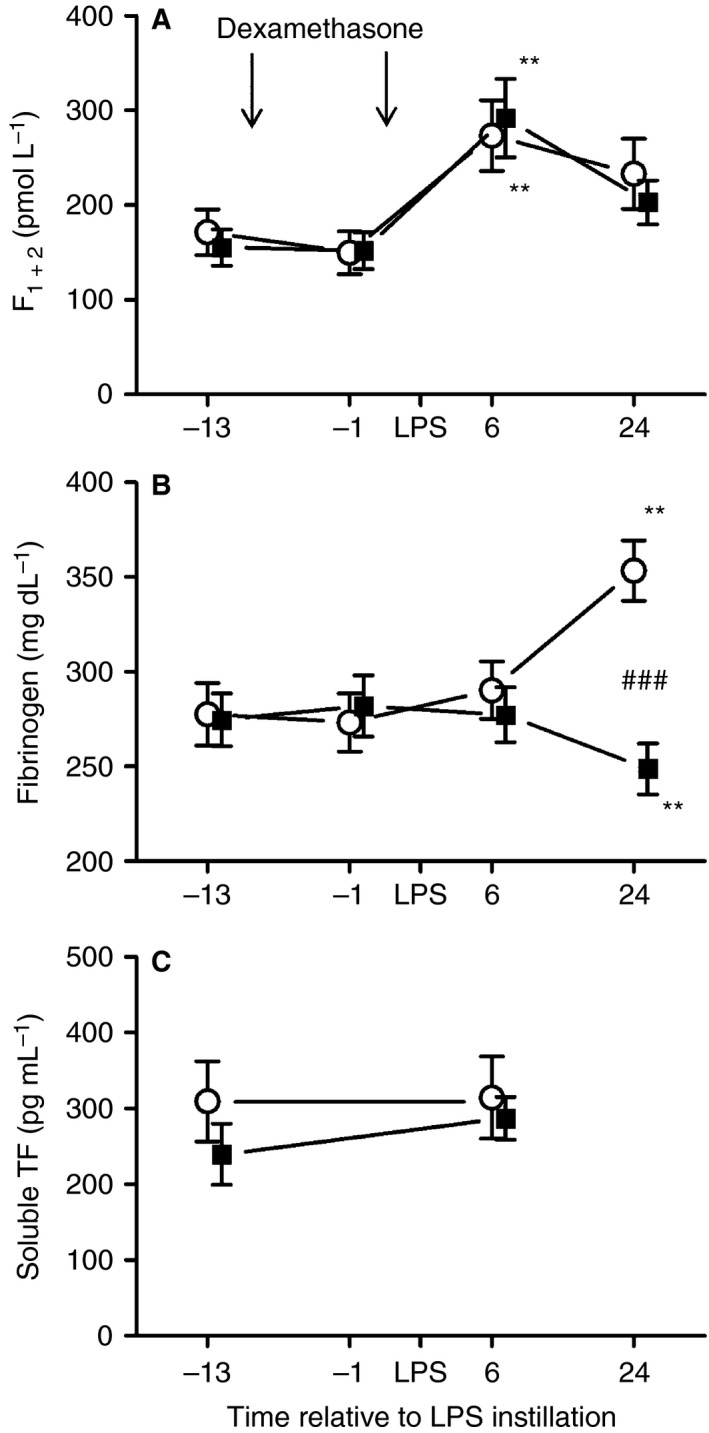
Prothrombin fragment 1 + 2 (F_1 + 2_) (A), fibrinogen (B) and soluble tissue factor (TF) (C) levels in plasma before and after bronchial lipopolysaccharide (LPS) (4 ng kg^−1^) challenge in healthy volunteers who received 2 × 40 mg of dexamethasone (■) (*n* = 11) or placebo (○) (*n* = 13). Venous blood samples were drawn before drug administration (13 h and 1 h before LPS challenge), and 6 h and 24 h after LPS challenge. Data represent means ± standard errors of the mean. **P < 0.01 versus baseline (13 h before LPS challenge); ###*P* < 0.001 for comparison between dexamethasone and placebo treatment.

Plasma levels of F_1 + 2_ increased slightly in both groups 6 h after LPS instillation as compared with baseline (*P* = 0.009 for placebo and *P* = 0.005 for dexamethasone; Fig. 3A). Plasma fibrinogen levels increased by 27% (*P* = 0.003 as compared with baseline values 13 h before LPS challenge; Fig. 3B) in the placebo group 24 h after LPS instillation. Dexamethasone decreased plasma fibrinogen levels by 10% (*P* = 0.004 as compared with baseline; Fig. 3B) 24 h after LPS instillation. Dexamethasone reduced fibrinogen levels by 30% (*P* < 0.001; Fig. 3B) as compared with placebo 24 h after LPS instillation. Plasma sTF levels ranged from 96 pg mL^−1^ to 884 pg mL^−1^; however, no significant difference was detectable (Fig. 3C).

## Discussion

Glucocorticoids are associated with an increased risk of thrombosis 12, but their effects on local pulmonary coagulation have not yet been studied. We therefore conducted a randomized, double‐blind, placebo‐controlled trial to characterize the effects of dexamethasone on pulmonary coagulation induced by endotoxin in healthy volunteers.

In the present study, instillation of 4 ng kg^−1^ LPS increased BALF levels of F_1 + 2_ three‐fold, a direct indicator of thrombin generation 25. This is in accordance with an observational study in which 29 patients with ventilator‐associated pneumonia had significantly higher F_1 + 2_ BALF levels than patients without pneumonia 26. Furthermore, we found significantly higher levels of TATc in LPS‐challenged lung sites, consistent with previous experimental studies 27, 28.

Dexamethasone suppressed the LPS‐enhanced thrombin generation to levels measured in BALF from the saline‐instilled segments in the placebo group. Interestingly, dexamethasone even lowered F_1 + 2_ and TATc levels in saline‐instilled segments as compared with placebo.

Inhibition of thrombin generation in the lung by dexamethasone is in contrast to the observed systemic increase in thrombin generation when prednisolone was given before intravenous LPS infusion 15. Ten days of prednisolone treatment also increased thrombin generation in healthy volunteers 13, and dexamethasone increased von Willebrand factor and soluble P‐selectin levels 29. *In vitro* studies have suggested that glucocorticoids increase LPS‐induced TF expression in human monocytes, which constitute the main source of TF in the circulation 30, 31. Despite a trend of higher sTF levels in LPS‐challenged lungs, this was not statistically significant as in previous investigations 28. Considering that constitutive TF expression is high in bronchi and in the lung 32, 33, it is possible that the high physiologic background noise may have interfered with the detection of a putative increase in the BALF TF content of LPS‐challenged segments. Although TF is certainly fundamental for the initiation of the extrinsic pathway, it is important to recognize that, in the lungs, TF, given its abundance, is not the rate‐limiting factor in the cascade 34.

Although dexamethasone inhibited procoagulant activity by 50% in cultured bovine alveolar macrophages 35, the mechanisms by which dexamethasone decreases thrombin generation *in vivo* are probably different. Thrombin generation in the bronchoalveolar compartment is dependent on clotting factors 34, which are thought to be mainly restricted to the plasma compartment. In our model, dexamethasone prevented the LPS‐induced protein extravasation into the lung compartment, and even reduced protein extravasation in saline‐treated lung segments 16. This suggests that reduced amounts of coagulation factors, including FII, are translocated from pulmonary capillaries into the bronchoalveolar compartment, thereby limiting the availability of substrate and therefore thrombin generation.

PAI‐1 activity and antigen levels were below the lower limit of quantification in 95% of BALF samples. Consequently, we could not reliably determine whether instillation of LPS or dexamethasone treatment influenced fibrinolysis in the lung.

Regarding the systemic effects of dexamethasone in our trial, dexamethasone suppressed plasma fibrinogen levels by 25%, which is a well‐known effect of glucocorticoids on acute‐phase reactants 14.

Plasma levels of F_1 + 2_ increased slightly after LPS instillation, without a difference between groups. Plasma levels of F_1 + 2_ are increased in patients with pneumonia 36 and in healthy volunteers after LPS infusion 37, 38.

Particular strengths of our study include the randomized, double‐blind and placebo‐controlled design, and the instillation of saline and LPS in the same subject, thereby reducing interindividual variability.

There are some limitations regarding the nature of the experimental design. A study population such as healthy volunteers is younger and is not necessarily representative of patients with a pulmonary infection admitted to the hospital. Such patients have more severe lung inflammation and multiple comorbidities that may influence the effects of glucocorticoids or, more likely, the disease course. The LPS‐induced procoagulant shift in the lung is lower than in patients suffering from, for example, pneumonia and has a self‐limiting course. Dexamethasone was given before LPS challenge, because it generates only a transient and self‐limiting lung inflammation. A sustained LPS stimulus is not feasible in healthy volunteers, because of ethical concerns. Thus, the applied design primarily allows for the investigation of prevention strategies.

One clear limitation is that we have only tested the prophylactic effects of dexamethasone, because glucocorticoids mainly work by inducing or inhibiting the mRNA transcription of various genes. A reasonable speculation is that the later one uses glucocorticoids in our model, the less effective they will be. This will possibly be different in a clinical situation such as pneumonia, where there is sustained inflammation that may lead to ARDS, so that glucocorticoids given later during the course of pneumonia may still prevent ARDS.

Our results indicate that systemic glucocorticoid therapy inhibits thrombin generation in the lung and may therefore limit pulmonary coagulopathy in patients. In a recent systematic review and meta‐analysis, glucocorticoid treatment was associated with a decreased risk of ARDS in patients with community‐acquired pneumonia (CAP) 39. Our findings provide a mechanistic explanation for how glucocorticoid therapy may prevent CAP patients from developing ARDS, in view of pulmonary fibrin deposition being characteristic for ARDS.

Glucocorticoid therapy given within 72 h after ARDS onset was associated with a reduction in the duration of mechanical ventilation, intensive care unit (ICU) stay and ICU mortality in a small, double‐blind trial 40. Whether glucocorticoid therapy is beneficial in early ARDS remains to be confirmed in larger trials 41, but our data allow for speculation that the observed reduction in vascular permeability may be followed by less bronchoalveolar coagulopathy in such patients. Overall, the present study provides insights into glucocorticoid action in the lung, and highlights the fact that glucocorticoids reduce coagulation in the pulmonary compartment.

## Addendum

J. Bartko, B. Jilma, J. Wojta, and L. Stiebellehner were responsible for the conception and design. J. Bartko, B. Jilma, C. Schoergenhofer, M. Schwameis, and N. Buchtele were responsible for analysis and/or interpretation. J. Bartko, B. Jilma, G. Schabbauer, and L. Stiebellehner wrote the manuscript. All authors gave final approval of the version to be published.

## Disclosure of Conflict of Interests

The authors state that they have no conflict of interest.

## Supporting information


**Table S1.** Sensitivity analysis for BALF recovery.

## References

[1] Milbrandt EB , Reade MC , Lee M , Shook SL , Angus DC , Kong L , Carter M , Yealy DM , Kellum JA . Prevalence and significance of coagulation abnormalities in community‐acquired pneumonia. Mol Med 2009; 15: 438–45. 19753144 10.2119/molmed.2009.00091 PMC2743205

[2] Gunther A , Mosavi P , Heinemann S , Ruppert C , Muth H , Markart P , Grimminger F , Walmrath D , Temmesfeld‐Wollbruck B , Seeger W . Alveolar fibrin formation caused by enhanced procoagulant and depressed fibrinolytic capacities in severe pneumonia. Comparison with the acute respiratory distress syndrome. Am J Respir Crit Care Med 2000; 161: 454–62. 10673185 10.1164/ajrccm.161.2.9712038

[3] Schultz MJ , Millo J , Levi M , Hack CE , Weverling GJ , Garrard CS , van der Poll T . Local activation of coagulation and inhibition of fibrinolysis in the lung during ventilator associated pneumonia. Thorax 2004; 59: 130–5. 14760153 10.1136/thorax.2003.013888 PMC1746934

[4] Rijneveld AW , Weijer S , Bresser P , Florquin S , Vlasuk GP , Rote WE , Spek CA , Reitsma PH , van der Zee JS , Levi M , van der Poll T . Local activation of the tissue factor–factor VIIa pathway in patients with pneumonia and the effect of inhibition of this pathway in murine pneumococcal pneumonia. Crit Care Med 2006; 34: 1725–30. 16625114 10.1097/01.CCM.0000218807.20570.C2

[5] Sebag SC , Bastarache JA , Ware LB . Therapeutic modulation of coagulation and fibrinolysis in acute lung injury and the acute respiratory distress syndrome. Curr Pharm Biotechnol 2011; 12: 1481–96. 21401517 10.2174/138920111798281171 PMC3893117

[6] Idell S . Extravascular coagulation and fibrin deposition in acute lung injury. New Horiz 1994; 2: 566–74. 7804805

[7] Headley AS , Tolley E , Meduri GU . Infections and the inflammatory response in acute respiratory distress syndrome. Chest 1997; 111: 1306–21. 9149588 10.1378/chest.111.5.1306

[8] Torres A , Sibila O , Ferrer M , Polverino E , Menendez R , Mensa J , Gabarrus A , Sellares J , Restrepo MI , Anzueto A , Niederman MS , Agusti C . Effect of corticosteroids on treatment failure among hospitalized patients with severe community‐acquired pneumonia and high inflammatory response: a randomized clinical trial. JAMA 2015; 313: 677–86. 25688779 10.1001/jama.2015.88

[9] Snijders D , Daniels JM , de Graaff CS , van der Werf TS , Boersma WG . Efficacy of corticosteroids in community‐acquired pneumonia: a randomized double‐blinded clinical trial. Am J Respir Crit Care Med 2010; 181: 975–82. 20133929 10.1164/rccm.200905-0808OC

[10] Meduri GU , Marik PE , Chrousos GP , Pastores SM , Arlt W , Beishuizen A , Bokhari F , Zaloga G , Annane D . Steroid treatment in ARDS: a critical appraisal of the ARDS network trial and the recent literature. Intensive Care Med 2008; 34: 61–9. 18000649 10.1007/s00134-007-0933-3

[11] Agarwal R , Nath A , Aggarwal AN , Gupta D . Do glucocorticoids decrease mortality in acute respiratory distress syndrome? A meta‐analysis. Respirology 2007; 12: 585–90. 17587427 10.1111/j.1440-1843.2007.01060.x

[12] Johannesdottir SA , Horvath‐Puho E , Dekkers OM , Cannegieter SC , Jorgensen JO , Ehrenstein V , Vandenbroucke JP , Pedersen L , Sorensen HT . Use of glucocorticoids and risk of venous thromboembolism: a nationwide population‐based case‐control study. JAMA Intern Med 2013; 173: 743–52. 23546607 10.1001/jamainternmed.2013.122

[13] Majoor CJ , Sneeboer MM , de Kievit A , Meijers JC , van der Poll T , Lutter R , Bel EH , Kamphuisen PW . The influence of corticosteroids on haemostasis in healthy subjects. J Thromb Haemost 2016; 14: 716–723. 26791678 10.1111/jth.13265

[14] van Zaane B , Nur E , Squizzato A , Gerdes VE , Buller HR , Dekkers OM , Brandjes DP . Systematic review on the effect of glucocorticoid use on procoagulant, anti‐coagulant and fibrinolytic factors. J Thromb Haemost 2010; 8: 2483–93. 20735729 10.1111/j.1538-7836.2010.04034.x

[15] de Kruif MD , Lemaire LC , Giebelen IA , van Zoelen MA , Pater JM , van den Pangaart PS , Groot AP , de Vos AF , Elliott PJ , Meijers JC , Levi M , van der Poll T . Prednisolone dose‐dependently influences inflammation and coagulation during human endotoxemia. J Immunol 2007; 178: 1845–51. 17237435 10.4049/jimmunol.178.3.1845

[16] Bartko J , Stiebellehner L , Derhaschnig U , Schoergenhofer C , Schwameis M , Prosch H , Jilma B . Dissociation between systemic and pulmonary anti‐inflammatory effects of dexamethasone in humans. Br J Clin Pharmacol 2016; 81: 865–77. 26647918 10.1111/bcp.12857 PMC4834593

[17] O'Grady NP , Preas HL , Pugin J , Fiuza C , Tropea M , Reda D , Banks SM , Suffredini AF . Local inflammatory responses following bronchial endotoxin instillation in humans. Am J Respir Crit Care Med 2001; 163: 1591–8. 11401879 10.1164/ajrccm.163.7.2009111

[18] Reynier F , de Vos AF , Hoogerwerf JJ , Bresser P , van der Zee JS , Paye M , Pachot A , Mougin B , van der Poll T . Gene expression profiles in alveolar macrophages induced by lipopolysaccharide in humans. Mol Med 2012; 18: 1303–11. 22952057 10.2119/molmed.2012.00230 PMC3521791

[19] Hoogerwerf JJ , de Vos AF , van't Veer C , Bresser P , de Boer A , Tanck MW , Draing C , van der Zee JS , van der Poll T . Priming of alveolar macrophages upon instillation of lipopolysaccharide in the human lung. Am J Respir Cell Mol Biol 2010; 42: 349–56. 19448156 10.1165/rcmb.2008-0362OC

[20] Gupta V , Banyard A , Mullan A , Sriskantharajah S , Southworth T , Singh D . Characterization of the inflammatory response to inhaled lipopolysaccharide in mild to moderate chronic obstructive pulmonary disease. Br J Clin Pharmacol 2015; 79: 767–76. 25377849 10.1111/bcp.12546 PMC4415713

[21] Kager LM , de Boer JD , Bresser P , van der Zee JS , Zeerleder S , Meijers JC , van‘t Veer C , van der Poll T . Intrabronchial activated protein C enhances lipopolysaccharide‐induced pulmonary responses. Eur Respir J 2013; 42: 188–97. 23060625 10.1183/09031936.00057112

[22] Maris NA , de Vos AF , Bresser P , van der Zee JS , Meijers JC , Lijnen HR , Levi M , Jansen HM , van der Poll T . Activation of coagulation and inhibition of fibrinolysis in the lung after inhalation of lipopolysaccharide by healthy volunteers. Thromb Haemost 2005; 93: 1036–40. 15968385 10.1160/TH04-08-0492

[23] Pernerstorfer T , Stohlawetz P , Hollenstein U , Dzirlo L , Eichler HG , Kapiotis S , Jilma B , Speiser W . Endotoxin‐induced activation of the coagulation cascade in humans: effect of acetylsalicylic acid and acetaminophen. Arterioscler Thromb Vasc Biol 1999; 19: 2517–23. 10521382 10.1161/01.atv.19.10.2517

[24] Leitner JM , Firbas C , Mayr FB , Reiter RA , Steinlechner B , Jilma B . Recombinant human antithrombin inhibits thrombin formation and interleukin 6 release in human endotoxemia. Clin Pharmacol Ther 2006; 79: 23–34. 16413239 10.1016/j.clpt.2005.10.003

[25] Pelzer H , Schwarz A , Stuber W . Determination of human prothrombin activation fragment 1 + 2 in plasma with an antibody against a synthetic peptide. Thromb Haemost 1991; 65: 153–9. 2053101

[26] El‐Solh AA , Okada M , Pietrantoni C , Aquilina A , Berbary E . Procoagulant and fibrinolytic activity in ventilator‐associated pneumonia: impact of inadequate antimicrobial therapy. Intensive Care Med 2004; 30: 1914–20. 15278268 10.1007/s00134-004-2391-5

[27] van der Poll T , Levi M , Nick JA , Abraham E . Activated protein C inhibits local coagulation after intrapulmonary delivery of endotoxin in humans. Am J Respir Crit Care Med 2005; 171: 1125–8. 15750041 10.1164/rccm.200411-1483OC PMC2718442

[28] Hoogerwerf JJ , de Vos AF , Levi M , Bresser P , van der Zee JS , Draing C , von Aulock S , van der Poll T . Activation of coagulation and inhibition of fibrinolysis in the human lung on bronchial instillation of lipoteichoic acid and lipopolysaccharide. Crit Care Med 2009; 37: 619–25. 19114879 10.1097/CCM.0b013e31819584f9

[29] Jilma B , Cvitko T , Winter‐Fabry A , Petroczi K , Quehenberger P , Blann AD . High dose dexamethasone increases circulating P‐selectin and von Willebrand factor levels in healthy men. Thromb Haemost 2005; 94: 797–801. 16270633 10.1160/TH04-10-0652

[30] Bottles KD , Morrissey JH . Dexamethasone enhances agonist induction of tissue factor in monocytes but not in endothelial cells. Blood Coagul Fibrinolysis 1993; 4: 405–14. 8329565 10.1097/00001721-199306000-00002

[31] Reddy KV , Bhattacharjee G , Schabbauer G , Hollis A , Kempf K , Tencati M , O'Connell M , Guha M , Mackman N . Dexamethasone enhances LPS induction of tissue factor expression in human monocytic cells by increasing tissue factor mRNA stability. J Leukoc Biol 2004; 76: 145–51. 15075360 10.1189/jlb.0204068

[32] Drake TA , Morrissey JH , Edgington TS . Selective cellular expression of tissue factor in human tissues. Implications for disorders of hemostasis and thrombosis. Am J Pathol 1989; 134: 1087–97. 2719077 PMC1879887

[33] Osterud B , Bjorklid E . Sources of tissue factor. Semin Thromb Hemost 2006; 32: 11–23. 16479458 10.1055/s-2006-933336

[34] Chapman HA , Stahl M , Allen CL , Yee R , Fair DS . Regulation of the procoagulant activity within the bronchoalveolar compartment of normal human lung. Am Rev Respir Dis 1988; 137: 1417–25. 3202380 10.1164/ajrccm/137.6.1417

[35] Car BD , Slauson DO , Suyemoto MM , Dore M , Neilsen NR . Expression and kinetics of induced procoagulant activity in bovine pulmonary alveolar macrophages. Exp Lung Res 1991; 17: 939–57. 1959504 10.3109/01902149109064327

[36] Langstrom S , Peltola V , Petaja J , Ruuskanen O , Heikinheimo M . Enhanced thrombin generation and depressed anticoagulant function in children with pneumonia. Acta Paediatr 2012; 101: 919–23. 22646857 10.1111/j.1651-2227.2012.02746.x

[37] Mayr FB , Spiel AO , Leitner JM , Firbas C , Jilma‐Stohlawetz P , Chang JY , Key NS , Jilma B . Racial differences in endotoxin‐induced tissue factor‐triggered coagulation. J Thromb Haemost 2009; 7: 634–40. 19187081 10.1111/j.1538-7836.2009.03307.x

[38] Schoergenhofer C , Schwameis M , Hobl EL , Ay C , Key NS , Derhaschnig U , Jilma B , Spiel AO . Potent irreversible P2Y12 inhibition does not reduce LPS‐induced coagulation activation in a randomized, double‐blind, placebo‐controlled trial. Clin Sci (Lond) 2016; 130: 433–40. 26554025 10.1042/CS20150591

[39] Wan YD , Sun TW , Liu ZQ , Zhang SG , Wang LX , Kan QC . Efficacy and safety of corticosteroids for community‐acquired pneumonia: a systematic review and meta‐analysis. Chest 2016; 149: 209–19. 26501852 10.1378/chest.15-1733

[40] Meduri GU , Golden E , Freire AX , Taylor E , Zaman M , Carson SJ , Gibson M , Umberger R . Methylprednisolone infusion in early severe ARDS: results of a randomized controlled trial. Chest 2007; 131: 954–63. 17426195 10.1378/chest.06-2100

[41] Peter JV , John P , Graham PL , Moran JL , George IA , Bersten A . Corticosteroids in the prevention and treatment of acute respiratory distress syndrome (ARDS) in adults: meta‐analysis. BMJ 2008; 336: 1006–9. 18434379 10.1136/bmj.39537.939039.BE PMC2364864

