# Supplementary material for: Dexamethasone inhibits endotoxin‐induced coagulopathy in human lungs
Source: J Thromb Haemost. 2016 Oct 24;14(12):2471–7. doi: 10.1111/jth.13504 (PMC5298044; doi:10.1111/jth.13504)
Supplement: Supplementary file 1 — Table S1. Sensitivity analysis for BALF recovery. [file JTH-14-2471-s001.docx]

| Prothrombin fragment 1+2 in BAL (pmol·L^-1^) | Saline | LPS | p-value saline vs LPS |
| --- | --- | --- | --- |
| **Placebo** |  |  |  |
| unadjusted | 248 | 743 | 0.007 |
| adjusted ^1^ | 87 | 299 | 0.011 |
| adjusted ^2^ | 483 | 1922 | 0.005 |
| **Dexamethasone** |  |  |  |
| unadjusted | 49 | 283 | 0.007 |
| adjusted ^1^ | 26 | 121 | 0.006 |
| adjusted ^2^ | 96 | 740 | 0.006 |
| p-value dexamethasone vs Placebo | 0.014 | 0.028 |  |
| p-value of the sensitivity analysis 1 | 0.012 | 0.046 |  |
| p-value of the sensitivity analysis 2 | 0.014 | 0.035 |  |
| ^1^ formula: Prothrombin fragment 1+2 x BAL-fluid volume recovered / lavage volume (=140ml) | | | |
| ^2^ formula: Prothrombin fragment 1+2 x lavage volume (=140ml) / BAL-fluid volume recovered | | | |
